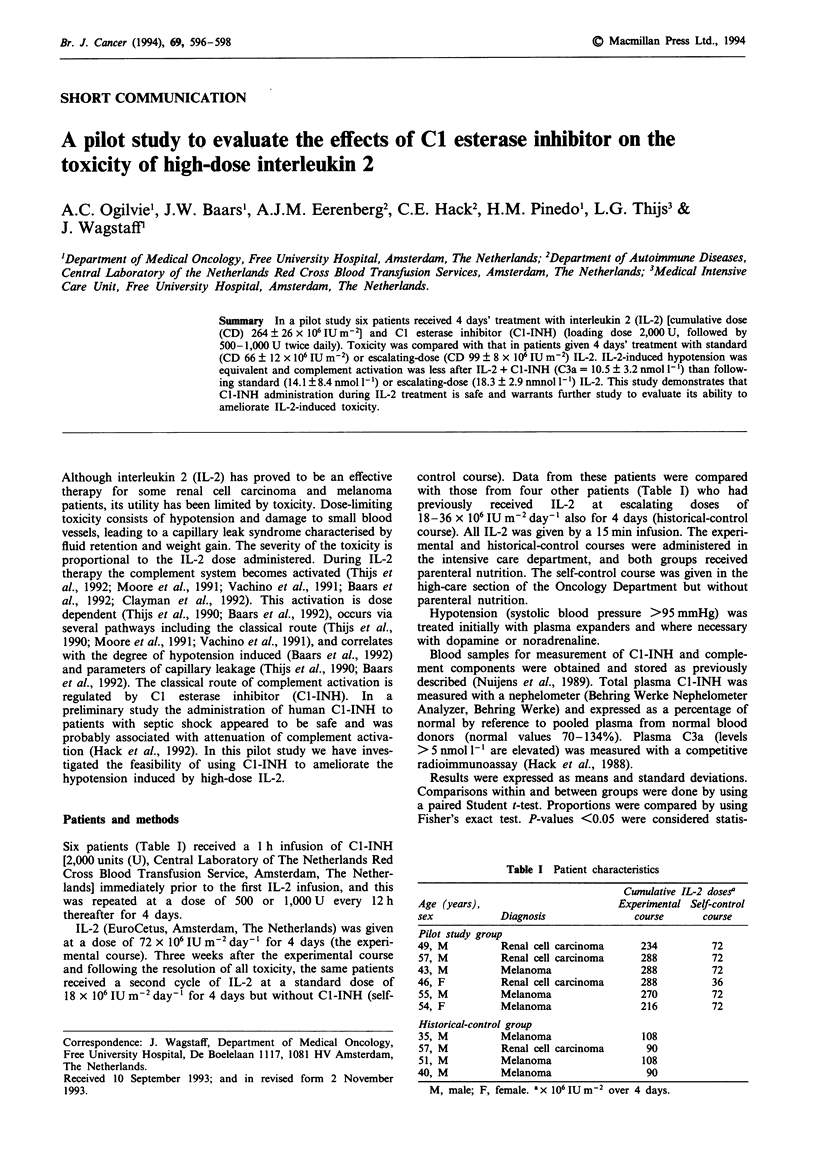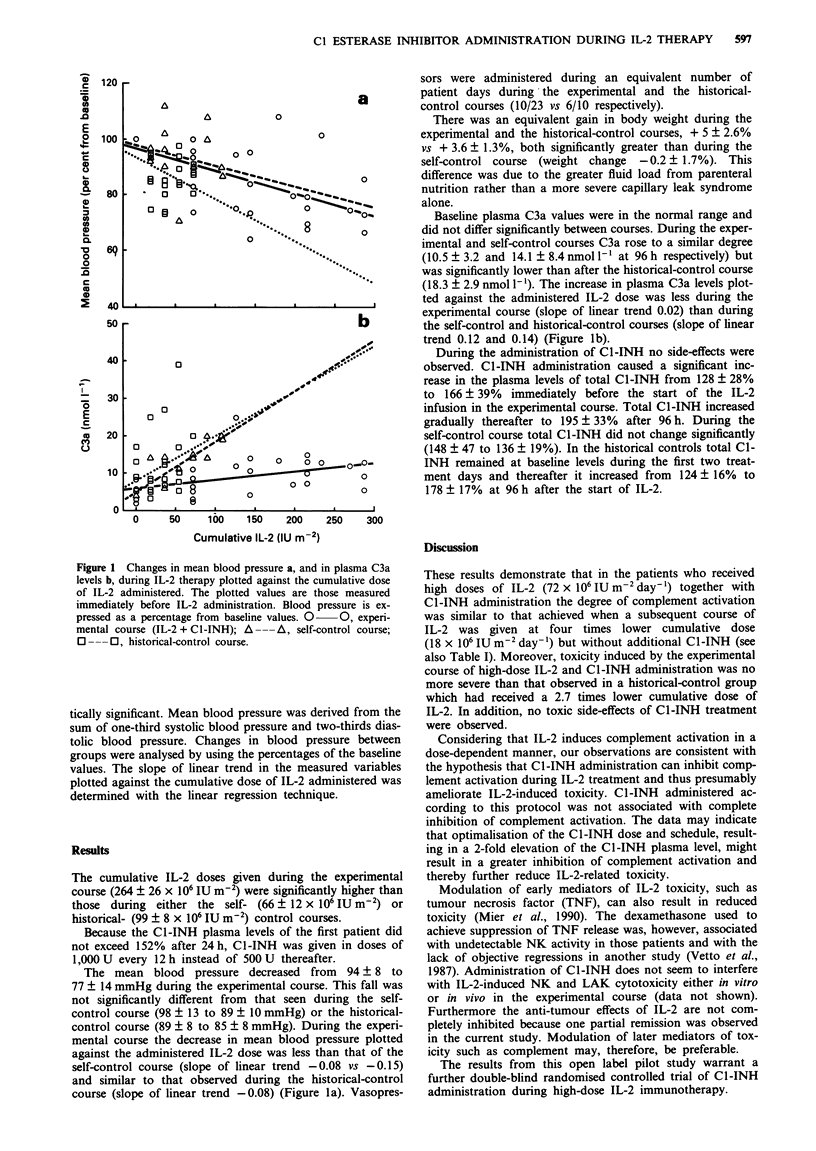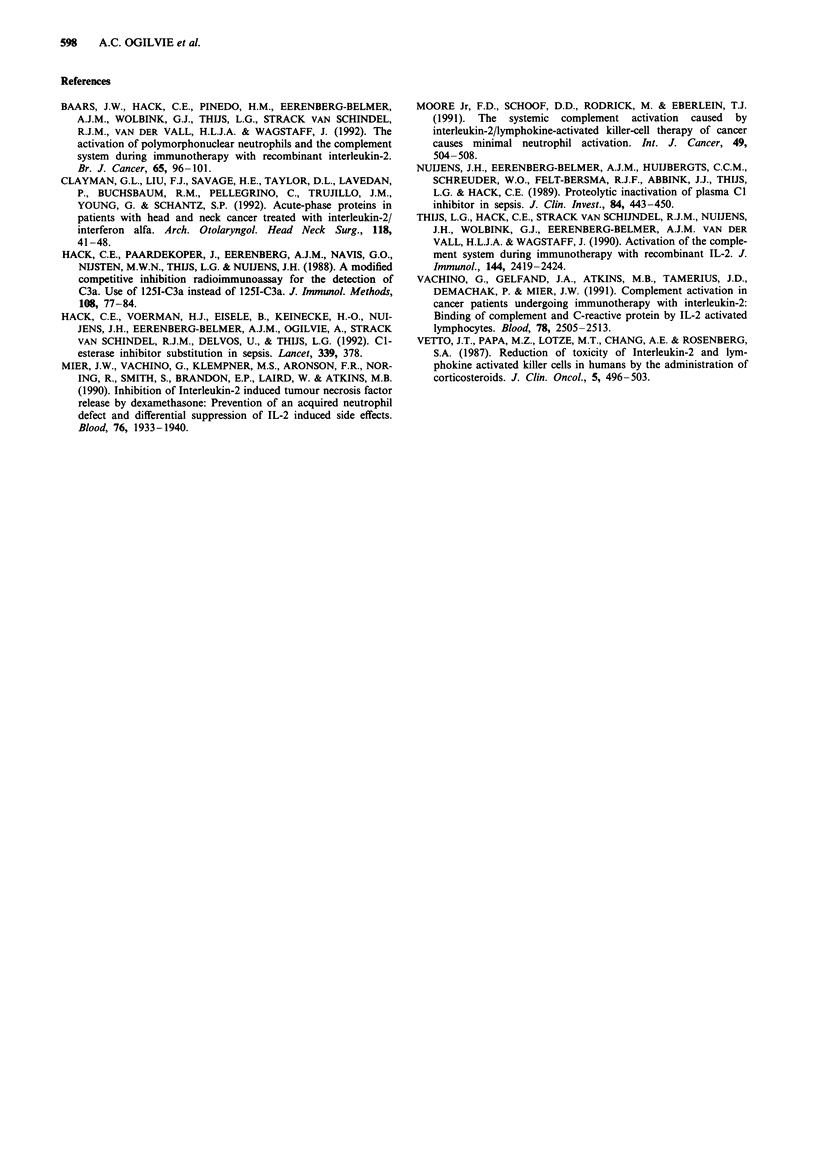# A pilot study to evaluate the effects of C1 esterase inhibitor on the toxicity of high-dose interleukin 2.

**DOI:** 10.1038/bjc.1994.109

**Published:** 1994-03

**Authors:** A. C. Ogilvie, J. W. Baars, A. J. Eerenberg, C. E. Hack, H. M. Pinedo, L. G. Thijs, J. Wagstaff

**Affiliations:** Department of Medical Oncology, Free University Hospital, Amsterdam, The Netherlands.

## Abstract

In a pilot study six patients received 4 days' treatment with interleukin 2 (IL-2) [cumulative dose (CD) 264 +/- 26 x 10(6) IU m-2] and C1 esterase inhibitor (C1-INH) (loading dose 2,000 U, followed by 500-1,000 U twice daily). Toxicity was compared with that in patients given 4 days' treatment with standard (CD 66 +/- 12 x 10(6) IU m-2) or escalating-dose (CD 99 +/- 8 x 10(6) IU m-2) IL-2. IL-2-induced hypotension was equivalent and complement activation was less after IL-2 + C1-INH (C3a = 10.5 +/- 3.2 nmol l-1) than following standard (14.1 +/- 8.4 nmol l-1) or escalating-dose (18.3 +/- 2.9 nmol l-1) IL-2. This study demonstrates that C1-INH administration during IL-2 treatment is safe and warrants further study to evaluate its ability to ameliorate IL-2-induced toxicity.


Br. J. Cancer (1994), 69, 596-598 © Macmillan Press Ltd., 1994

SHORT COMMUNICATION

### A pilot study to evaluate the effects of C1 esterase inhibitor on the toxicity of high-dose interleukin 2

A.C. Ogilvie[1], J.W. Baars[1], A.J.M. Eerenberg[2], C.E. Hack[2], H.M. Pinedo[1], L.G. Thijs[3] & J. Wagstaff[1]

[1]Department of Medical Oncology, Free University Hospital, Amsterdam, The Netherlands; [2]Department of Autoimmune Diseases, Central Laboratory of the Netherlands Red Cross Blood Transfusion Services, Amsterdam, The Netherlands; [3]Medical Intensive Care Unit, Free University Hospital, Amsterdam, The Netherlands.

**Summary** In a pilot study six patients received 4 days' treatment with interleukin 2 (IL-2) [cumulative dose (CD) $264 \pm 26 \times 10^6$ IU m$^{-2}$] and C1 esterase inhibitor (C1-INH) (loading dose 2,000 U, followed by 500-1,000 U twice daily). Toxicity was compared with that in patients given 4 days' treatment with standard (CD $66 \pm 12 \times 10^6$ IU m$^{-2}$) or escalating-dose (CD $99 \pm 8 \times 10^6$ IU m$^{-2}$) IL-2. IL-2-induced hypotension was equivalent and complement activation was less after IL-2 + C1-INH (C3a = $10.5 \pm 3.2$ nmol l$^{-1}$) than following standard ($14.1 \pm 8.4$ nmol l$^{-1}$) or escalating-dose ($18.3 \pm 2.9$ nmnol l$^{-1}$) IL-2. This study demonstrates that C1-INH administration during IL-2 treatment is safe and warrants further study to evaluate its ability to ameliorate IL-2-induced toxicity.

Although interleukin 2 (IL-2) has proved to be an effective therapy for some renal cell carcinoma and melanoma patients, its utility has been limited by toxicity. Dose-limiting toxicity consists of hypotension and damage to small blood vessels, leading to a capillary leak syndrome characterised by fluid retention and weight gain. The severity of the toxicity is proportional to the IL-2 dose administered. During IL-2 therapy the complement system becomes activated (Thijs *et al.*, 1992; Moore *et al.*, 1991; Vachino *et al.*, 1991; Baars *et al.*, 1992; Clayman *et al.*, 1992). This activation is dose dependent (Thijs *et al.*, 1990; Baars *et al.*, 1992), occurs via several pathways including the classical route (Thijs *et al.*, 1990; Moore *et al.*, 1991; Vachino *et al.*, 1991), and correlates with the degree of hypotension induced (Baars *et al.*, 1992) and parameters of capillary leakage (Thijs *et al.*, 1990; Baars *et al.*, 1992). The classical route of complement activation is regulated by C1 esterase inhibitor (C1-INH). In a preliminary study the administration of human C1-INH to patients with septic shock appeared to be safe and was probably associated with attenuation of complement activation (Hack *et al.*, 1992). In this pilot study we have investigated the feasibility of using C1-INH to ameliorate the hypotension induced by high-dose IL-2.

### Patients and methods

Six patients (Table I) received a 1 h infusion of C1-INH [2,000 units (U), Central Laboratory of The Netherlands Red Cross Blood Transfusion Service, Amsterdam, The Netherlands] immediately prior to the first IL-2 infusion, and this was repeated at a dose of 500 or 1,000 U every 12 h thereafter for 4 days.

IL-2 (EuroCetus, Amsterdam, The Netherlands) was given at a dose of $72 \times 10^6$ IU m$^{-2}$ day$^{-1}$ for 4 days (the experimental course). Three weeks after the experimental course and following the resolution of all toxicity, the same patients received a second cycle of IL-2 at a standard dose of $18 \times 10^6$ IU m$^{-2}$ day$^{-1}$ for 4 days but without C1-INH (self-control course). Data from these patients were compared with those from four other patients (Table I) who had previously received IL-2 at escalating doses of $18-36 \times 10^6$ IU m$^{-2}$ day$^{-1}$ also for 4 days (historical-control course). All IL-2 was given by a 15 min infusion. The experimental and historical-control courses were administered in the intensive care department, and both groups received parenteral nutrition. The self-control course was given in the high-care section of the Oncology Department but without parenteral nutrition.

Hypotension (systolic blood pressure >95 mmHg) was treated initially with plasma expanders and where necessary with dopamine or noradrenaline.

Blood samples for measurement of C1-INH and complement components were obtained and stored as previously described (Nuijens *et al.*, 1989). Total plasma C1-INH was measured with a nephelometer (Behring Werke Nephelometer Analyzer, Behring Werke) and expressed as a percentage of normal by reference to pooled plasma from normal blood donors (normal values 70-134%). Plasma C3a (levels >5 nmol l$^{-1}$ are elevated) was measured with a competitive radioimmunoassay (Hack *et al.*, 1988).

Results were expressed as means and standard deviations. Comparisons within and between groups were done by using a paired Student *t*-test. Proportions were compared by using Fisher's exact test. *P*-values <0.05 were considered statis-

**Table I** Patient characteristics

| *Age (years), sex* | *Diagnosis* | *Cumulative IL-2 doses[a]* *Experimental course* | *Self-control course* |
|---|---|---|---|
| *Pilot study group* | | | |
| 49, M | Renal cell carcinoma | 234 | 72 |
| 57, M | Renal cell carcinoma | 288 | 72 |
| 43, M | Melanoma | 288 | 72 |
| 46, F | Renal cell carcinoma | 288 | 36 |
| 55, M | Melanoma | 270 | 72 |
| 54, F | Melanoma | 216 | 72 |
| *Historical-control group* | | | |
| 35, M | Melanoma | 108 | |
| 57, M | Renal cell carcinoma | 90 | |
| 51, M | Melanoma | 108 | |
| 40, M | Melanoma | 90 | |

M, male; F, female. [a]$\times 10^6$ IU m$^{-2}$ over 4 days.

Correspondence: J. Wagstaff, Department of Medical Oncology, Free University Hospital, De Boelelaan 1117, 1081 HV Amsterdam, The Netherlands.
Received 10 September 1993; and in revised form 2 November 1993.

**Figure 1** Changes in mean blood pressure **a**, and in plasma C3a levels **b**, during IL-2 therapy plotted against the cumulative dose of IL-2 administered. The plotted values are those measured immediately before IL-2 administration. Blood pressure is expressed as a percentage from baseline values. O——O, experimental course (IL-2 + C1-INH); Δ---Δ, self-control course; □---□, historical-control course.

tically significant. Mean blood pressure was derived from the sum of one-third systolic blood pressure and two-thirds diastolic blood pressure. Changes in blood pressure between groups were analysed by using the percentages of the baseline values. The slope of linear trend in the measured variables plotted against the cumulative dose of IL-2 administered was determined with the linear regression technique.

### Results

The cumulative IL-2 doses given during the experimental course ($264 \pm 26 \times 10^6$ IU m$^{-2}$) were significantly higher than those during either the self- ($66 \pm 12 \times 10^6$ IU m$^{-2}$) or historical- ($99 \pm 8 \times 10^6$ IU m$^{-2}$) control courses.

Because the C1-INH plasma levels of the first patient did not exceed 152% after 24 h, C1-INH was given in doses of 1,000 U every 12 h instead of 500 U thereafter.

The mean blood pressure decreased from $94 \pm 8$ to $77 \pm 14$ mmHg during the experimental course. This fall was not significantly different from that seen during the self-control course ($98 \pm 13$ to $89 \pm 10$ mmHg) or the historical-control course ($89 \pm 8$ to $85 \pm 8$ mmHg). During the experimental course the decrease in mean blood pressure plotted against the administered IL-2 dose was less than that of the self-control course (slope of linear trend −0.08 *vs* −0.15) and similar to that observed during the historical-control course (slope of linear trend −0.08) (Figure 1a). Vasopressors were administered during an equivalent number of patient days during the experimental and the historical-control courses (10/23 *vs* 6/10 respectively).

There was an equivalent gain in body weight during the experimental and the historical-control courses, $+5 \pm 2.6\%$ *vs* $+3.6 \pm 1.3\%$, both significantly greater than during the self-control course (weight change $-0.2 \pm 1.7\%$). This difference was due to the greater fluid load from parenteral nutrition rather than a more severe capillary leak syndrome alone.

Baseline plasma C3a values were in the normal range and did not differ significantly between courses. During the experimental and self-control courses C3a rose to a similar degree ($10.5 \pm 3.2$ and $14.1 \pm 8.4$ nmol l$^{-1}$ at 96 h respectively) but was significantly lower than after the historical-control course ($18.3 \pm 2.9$ nmol l$^{-1}$). The increase in plasma C3a levels plotted against the administered IL-2 dose was less during the experimental course (slope of linear trend 0.02) than during the self-control and historical-control courses (slope of linear trend 0.12 and 0.14) (Figure 1b).

During the administration of C1-INH no side-effects were observed. C1-INH administration caused a significant increase in the plasma levels of total C1-INH from $128 \pm 28\%$ to $166 \pm 39\%$ immediately before the start of the IL-2 infusion in the experimental course. Total C1-INH increased gradually thereafter to $195 \pm 33\%$ after 96 h. During the self-control course total C1-INH did not change significantly ($148 \pm 47$ to $136 \pm 19\%$). In the historical controls total C1-INH remained at baseline levels during the first two treatment days and thereafter it increased from $124 \pm 16\%$ to $178 \pm 17\%$ at 96 h after the start of IL-2.

### Discussion

These results demonstrate that in the patients who received high doses of IL-2 ($72 \times 10^6$ IU m$^{-2}$ day$^{-1}$) together with C1-INH administration the degree of complement activation was similar to that achieved when a subsequent course of IL-2 was given at four times lower cumulative dose ($18 \times 10^6$ IU m$^{-2}$ day$^{-1}$) but without additional C1-INH (see also Table I). Moreover, toxicity induced by the experimental course of high-dose IL-2 and C1-INH administration was no more severe than that observed in a historical-control group which had received a 2.7 times lower cumulative dose of IL-2. In addition, no toxic side-effects of C1-INH treatment were observed.

Considering that IL-2 induces complement activation in a dose-dependent manner, our observations are consistent with the hypothesis that C1-INH administration can inhibit complement activation during IL-2 treatment and thus presumably ameliorate IL-2-induced toxicity. C1-INH administered according to this protocol was not associated with complete inhibition of complement activation. The data may indicate that optimalisation of the C1-INH dose and schedule, resulting in a 2-fold elevation of the C1-INH plasma level, might result in a greater inhibition of complement activation and thereby further reduce IL-2-related toxicity.

Modulation of early mediators of IL-2 toxicity, such as tumour necrosis factor (TNF), can also result in reduced toxicity (Mier *et al.*, 1990). The dexamethasone used to achieve suppression of TNF release was, however, associated with undetectable NK activity in those patients and with the lack of objective regressions in another study (Vetto *et al.*, 1987). Administration of C1-INH does not seem to interfere with IL-2-induced NK and LAK cytotoxicity either *in vitro* or *in vivo* in the experimental course (data not shown). Furthermore the anti-tumour effects of IL-2 are not completely inhibited because one partial remission was observed in the current study. Modulation of later mediators of toxicity such as complement may, therefore, be preferable.

The results from this open label pilot study warrant a further double-blind randomised controlled trial of C1-INH administration during high-dose IL-2 immunotherapy.

### References

BAARS, J.W., HACK, C.E., PINEDO, H.M., EERENBERG-BELMER, A.J.M., WOLBINK, G.J., THIJS, L.G., STRACK VAN SCHINDEL, R.J.M., VAN DER VALL, H.L.J.A. & WAGSTAFF, J. (1992). The activation of polymorphonuclear neutrophils and the complement system during immunotherapy with recombinant interleukin-2. *Br. J. Cancer*, **65**, 96–101.

CLAYMAN, G.L., LIU, F.J., SAVAGE, H.E., TAYLOR, D.L., LAVEDAN, P., BUCHSBAUM, R.M., PELLEGRINO, C., TRUJILLO, J.M., YOUNG, G. & SCHANTZ, S.P. (1992). Acute-phase proteins in patients with head and neck cancer treated with interleukin-2/interferon alfa. *Arch. Otolaryngol. Head Neck Surg.*, **118**, 41–48.

HACK, C.E., PAARDEKOPER, J., EERENBERG, A.J.M., NAVIS, G.O., NIJSTEN, M.W.N., THIJS, L.G. & NUIJENS, J.H. (1988). A modified competitive inhibition radioimmunoassay for the detection of C3a. Use of 125I-C3a instead of 125I-C3a. *J. Immunol. Methods*, **108**, 77–84.

HACK, C.E., VOERMAN, H.J., EISELE, B., KEINECKE, H.-O., NUIJENS, J.H., EERENBERG-BELMER, A.J.M., OGILVIE, A., STRACK VAN SCHINDEL, R.J.M., DELVOS, U., & THIJS, L.G. (1992). C1-esterase inhibitor substitution in sepsis. *Lancet*, **339**, 378.

MIER, J.W., VACHINO, G., KLEMPNER, M.S., ARONSON, F.R., NORING, R., SMITH, S., BRANDON, E.P., LAIRD, W. & ATKINS, M.B. (1990). Inhibition of Interleukin-2 induced tumour necrosis factor release by dexamethasone: Prevention of an acquired neutrophil defect and differential suppression of IL-2 induced side effects. *Blood*, **76**, 1933–1940.

MOORE Jr, F.D., SCHOOF, D.D., RODRICK, M. & EBERLEIN, T.J. (1991). The systemic complement activation caused by interleukin-2/lymphokine-activated killer-cell therapy of cancer causes minimal neutrophil activation. *Int. J. Cancer*, **49**, 504–508.

NUIJENS, J.H., EERENBERG-BELMER, A.J.M., HUIJBERGTS, C.C.M., SCHREUDER, W.O., FELT-BERSMA, R.J.F., ABBINK, J.J., THIJS, L.G. & HACK, C.E. (1989). Proteolytic inactivation of plasma C1 inhibitor in sepsis. *J. Clin. Invest.*, **84**, 443–450.

THIJS, L.G., HACK, C.E., STRACK VAN SCHIJNDEL, R.J.M., NUIJENS, J.H., WOLBINK, G.J., EERENBERG-BELMER, A.J.M. VAN DER VALL, H.L.J.A. & WAGSTAFF, J. (1990). Activation of the complement system during immunotherapy with recombinant IL-2. *J. Immunol.*, **144**, 2419–2424.

VACHINO, G., GELFAND, J.A., ATKINS, M.B., TAMERIUS, J.D., DEMACHAK, P. & MIER, J.W. (1991). Complement activation in cancer patients undergoing immunotherapy with interleukin-2: Binding of complement and C-reactive protein by IL-2 activated lymphocytes. *Blood*, **78**, 2505–2513.

VETTO, J.T., PAPA, M.Z., LOTZE, M.T., CHANG, A.E. & ROSENBERG, S.A. (1987). Reduction of toxicity of Interleukin-2 and lymphokine activated killer cells in humans by the administration of corticosteroids. *J. Clin. Oncol.*, **5**, 496–503.